# Sexual selection on population-level mating opportunities drives morph ratios in a fig wasp with extreme male dimorphism

**DOI:** 10.1186/s12862-021-01898-3

**Published:** 2021-09-06

**Authors:** James M. Cook

**Affiliations:** Hawkesbury Institute for the Environment, Western Sydney University, Locked Bag 1797, Penrith, NSW 2751 Australia

**Keywords:** Mating systems, Sexual selection, Fig wasp, Alternative strategies, Male polymorphism

## Abstract

**Background:**

Alternative mating tactics are widespread in animals and associated with extreme morphological polymorphism in some insects. Some fig wasps have both highly modified wingless males and dispersing winged males. Wingless males mate inside figs before females disperse, while winged males mate elsewhere after dispersal. Hamilton proposed a model for this system with morphs determined by alternative alleles. This has an equilibrium where the proportion of winged males equals the proportion of females dispersing unmated; i.e. the proportion of matings that they obtain. Previously, we have shown qualitative support for this prediction across nine wing-dimorphic fig wasp species. Here I test the quantitative prediction in the fig wasp *Pseudidarnes minerva*. In addition, some fig wasp species that lack winged males, but have two wingless morphs, show a conditional strategy with morph determination influenced by the number of wasps developing in a patch. I also test for this alternative pattern in the wing-dimorphic *P. minerva.*

**Results:**

I sampled 114 figs that contained a mean of 2.1 *P. minerva* wasps from 44 trees across four sites in Sydney, Australia. At the whole population level, the proportion of winged males (0.84 or 0.79 corrected for sampling bias) did not differ significantly from the proportion of unmated females (0.84), providing strong quantitative support for the prediction of Hamilton’s model. In addition, there was no evidence for other factors, such as local mate competition or fighting between wingless males, that could violate simplifying assumptions of the model. Meanwhile, the proportion of winged males was not correlated with the number of wasps per fig, providing no evidence for a conditional strategy.

**Conclusion:**

The morph ratio in *P. minerva* is consistent with Hamilton’s simple Mendelian strategy model, where morph ratios are set by average mating opportunities at the population level. This contrasts with some fig wasps from another subfamily that show conditional morph determination, allowing finer scale adaptation to fig-level mating opportunities. However, these conditional cases do not involve wing polymorphism. Male polymorphism is common and variable in fig wasps and has evolved independently in multiple lineages with apparently different underlying mechanisms.

**Supplementary Information:**

The online version contains supplementary material available at 10.1186/s12862-021-01898-3.

## Background

Sexual selection is a pervasive force that can drive dramatic phenotypic diversity both between and within the sexes [1, 2]. Alternative mating tactics within a sex are known in many animal species [2–5] and, in many cases, different behavioural tactics are matched by morphologies that are also distinct [2, 3]. The strategies underlying such polymorphism have been much debated (e.g. [2–6]) and two general types of models have been proposed. The first involves a simple genetic mechanism, involving alternative alleles at one or a few loci, where different phenotypes arise from different genotypes [7, 8]. This model requires morphs to have equal fitness to coexist, since otherwise the fitter strategy would become fixed, and predicts that the equilibrium proportion of each strategy equals the proportion of offspring that it produces [7–9]. To date there are relatively few good cases for species conforming to the alternative alleles model, but these involve diverse taxa. For example, amongst vertebrates, genetic male morphs with different mating behaviours occur in a bird [10], a lizard [11] and a fish [12]. Among invertebrates a key example is the marine isopod, *Paracerceis sculpta,* which has three male morphs determined by alternative alleles [13] for which there is good evidence for equal average fitness [2, 14]. Examples from insects include the damselfly *Mnais costalis* [15] and most recently, a weta, *Hemideina crassidens* (Orthoptera) [16]. Most known examples involve male polymorphism, but female genetic morphs occur in *Ischnura* damselflies and appear to be widespread in damselflies and dragonflies [17].

In contrast to the above, there are numerous examples of the second form of morph determination, where the phenotype expressed is a conditional strategy that depends on environmental or social cues [2, 3, 5]. For example, the mite *Rhizoglyphus echinopus* has a large fighting and a small non-fighting male morph and their relative proportions are determined by colony size [18]. However, conditional strategies can also depend on genetic effects, since most are thought to be threshold traits, where a key continuous variable (e.g. the level of a hormone or juvenile growth rate) determines the morph produced [6, 19]. Such threshold traits are generally underpinned by many genes with small effect, so the threshold can evolve adaptively to match local sexual selection pressures [6, 19, 20]. For example, further studies of *R. echinopus,* using an experimental evolution approach, have shown that the switch point between production of sneaker and fighter male mites can be changed by manipulating habitat complexity [20]. Importantly, if conditional morph determination responds to local patch conditions, adaptation can be more fine-grained than with a Mendelian strategy, where morph ratios may only be optimized at the coarser population level [2, 6, 21]. Consequently, some authors have argued that Mendelian strategies should be rare since they will generally be outcompeted by a conditional strategy with an evolving threshold [6]. Together, the theoretical basis and empirical evidence for the prevalence of conditional versus Mendelian strategies have formed a long-standing area of ongoing debate.

**Fig. 1:**
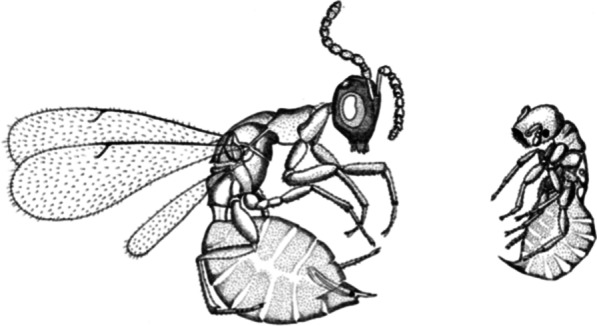
The winged and wingless male morphs of the fig wasp *Pseudidarnes minerva* provide an example of extreme male dimorphism. The original figure from Cook et al. [22] was drawn by Joanne Martin

Some of the most extreme cases of male polymorphism occur in fig wasps [8, 22–24], tiny insects that develop inside the inflorescences (figs) of *Ficus* trees. Many species have only winged males, which mate with females outside of figs (e.g. on fig leaves) after dispersal. These species typically have very few individuals developing per fig (hereafter brood size). In contrast, other species have only highly modified wingless males, adapted to searching for and mating with females inside the dark confines of the fig fruit [8, 22, 24, 25]. These species have large brood sizes (tens or hundreds of wasps developing per fig) and mating occurs inside the fig before the females disperse. Between these extremes, lie some species with intermediate brood sizes, in which winged and wingless male morphs coexist (see Fig. 1). In these wing-dimorphic species, wingless males mate with females inside figs, while winged males exit figs and mate with unmated females after dispersal. Females either mate with males inside their natal fig or leave the fig and disperse to mate with males elsewhere. Hamilton [8] predicted that male dimorphism would persist only in species with intermediate (and/or highly variable) brood sizes, which was supported by a subsequent comparative study [24].

While comparative analyses supports the general correlation between brood size and the existence of winged and/or wingless males across species [8, 24], no studies have tested quantitative predictions for morph ratios within a wing-dimorphic species. Hamilton [8] proposed a simple model for these male-haploid species, involving a single locus with alternative alleles for winged and wingless males. Wingless male fitness derives from pre-dispersal mating inside figs and winged male fitness from post-dispersal mating outside figs. Allele (and morph) frequencies therefore depend on the relative frequency of pre- and post-dispersal mating opportunities [8]. An equilibrium should occur when the frequency of winged males equals the frequency of females dispersing from their figs unmated. If winged males are less common than this, then they have more mating opportunities (higher fitness) and should increase in frequency. In contrast, if winged males are more common, they will have fewer per capita mating opportunities (lower fitness) and should decrease in frequency. Consequently, if we can measure the proportion of females dispersing unmated, we can test if this equals the proportion of winged males. In fig wasps, the former can be estimated by assuming that females developing in a fig with wingless males are mated by those males, and then assessing how many females develop in figs that do not contain wingless males [8, 23]. Meanwhile, the latter can be estimated from a large sample of males from the same population.

**Fig. 2:**
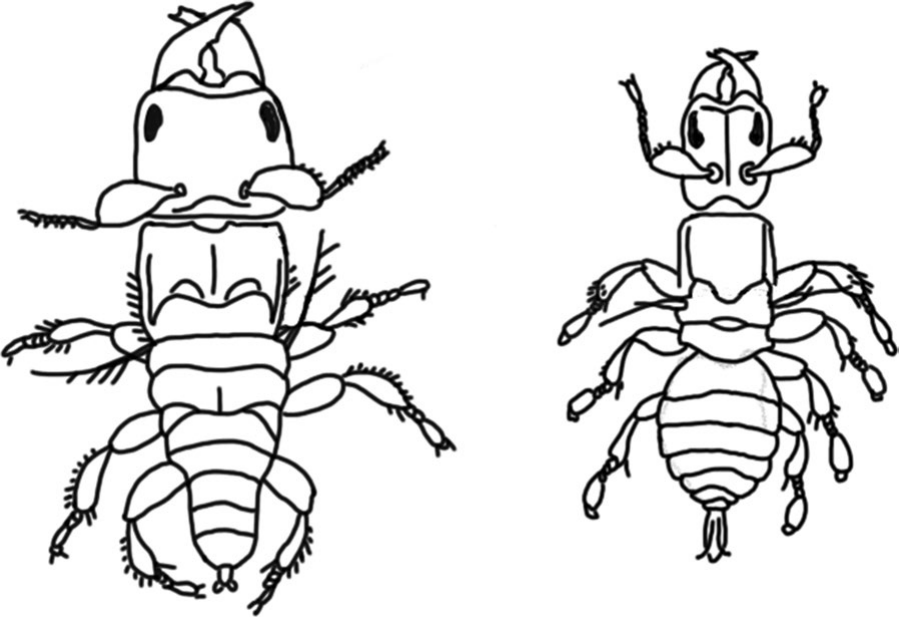
Less extreme male dimorphism in *Otitesella* species—both morphs are wingless. The larger, more combative *digitata* morph (left) and the smaller, more dispersive, *religiosa* morph (right). Drawings by JMC based on Westwood (1883)

To date, within-species studies of fig wasp male polymorphism have focused mainly on species with more nuanced dimorphisms (see Fig. 2) involving two (or more) types of wingless male [21, 26–30]. Interestingly, there is good evidence for conditional morph determination, linked to brood size, in two such wasp species from different genera (*Otitesella* and *Walkerella*) in the subfamily Otitesellinae [21, 27, 30]*.* Further, Pienaar & Greeff [21] pointed out that evidence across species for frequency-dependent morph ratios does not discriminate between genetic or conditional morph determination within species. Fig wasp life cycles can involve large fluctuations between generations in the proportion of females dispersing unmated and genetic morph determination would lead to a poor fit between morphs and mating opportunities in an individual fig or a crop of figs on a tree. Pienaar & Greeff [21, 27] showed that this fit in *Otitesella pseudoserrata* was too good to be consistent with alternative alleles, suggesting a conditional strategy. They further argued that temporal fluctuations in mating opportunities make it unlikely that genetic morph determination could persist in other fig wasps, but it seems premature to rule it out in general for the following reasons.

First, male polymorphism has evolved independently in several fig wasp lineages [24, 31–33], leading to hundreds of species with male polymorphism [23], and some may have genetically determined morphs [23]. Second, the cases discussed in the paragraph above do not involve wing dimorphism, only the more subtle differences between wingless male morphs (Fig. 2). Detection of different wingless male morphs is sometimes easy, but in other cases can require detailed quantitative analysis to reveal differences in the allometry of body parts [26, 29, 34]. It is therefore easy to imagine that the genetic and molecular mechanisms responsible for this type of variation (Fig. 2) are different to those driving the striking major differences between winged and wingless males (Fig. 1). Third, beyond fig wasps, there are examples of genetically determined male morphs in diverse vertebrate and invertebrate taxa (e.g. [10, 11, 13, 15, 16]).

In this study, I conduct the first detailed single species study of natural morph ratios and morph determination in a fig wasp species that is wing-dimorphic—*Pseudidarnes minerva* (Fig. 1) associated with *Ficus rubiginosa* trees in Australia. This species was included as a single data point in a previous comparative analysis of wing dimorphism across fig wasp species [24]. The proportion of winged males (0.77) was significantly higher than the estimated proportion of females dispersing unmated (0.42), but the data were a by-product of a community ecology study [35], with associated sampling limitations for the study of male dimorphism (see discussion). Here, I use a new data set in which wasps were sampled from multiple trees, sites and timepoints. This provides more appropriate sampling to (1) estimate and compare the proportion of winged males and females dispersing unmated at the population level; and (2) test if the proportion of wingless males increases with the number of wasps in a fig, suggesting a conditional strategy that allows fine-scale adaptation.

## Methods

### Sampling

In most monoecious fig species, individual trees flower asynchronously and sporadically, such that there is year-round flower and fruit production at the population level [36]. Moreover, in *F. rubiginosa*, individual trees at any one time may have figs at all stages of development from receptive to ripe [35]. From Nov 2013 to Dec 2015, monthly sampling was conducted from *F. rubignosa* trees at four sites in Greater Sydney—Manly, Balmoral, Penrith, Wisemans Ferry. The target sample size was > 50 figs pooled from > 4 trees per site, but due to the phenology described above, there were sampling sessions when either the number of trees in fruit or the number of ripe fruits was lower. Fruiting trees varied in height from about 2–10 m and figs were collected from the ground by hand or using pole pruners, allowing collection up to about 3 m. For many trees, this represented essentially the whole tree, but for large trees only some of the canopy was accessible.

Figs were collected when wasps were about to emerge from the fruits; i.e. when figs are yellow and slightly soft. The wasp species fall into two distinct size categories—large and small [37]. Large wasps, including *P. minerva,* can only exit figs through holes that they chew themselves, not through the very small holes made by the numerous pollinator wasps. It is therefore easy to tell whether any large wasps, such as *P. minerva,* have already emerged. Based on this, we excluded any figs that already had large holes when they were collected, since *P. minerva* wasps may have already dispersed from these samples. The remaining individual figs were placed in 70 ml plastic pots with gauze-covered tops (to prevent excess humidity and fungal growth) to allow recording of all wasps emerging. After allowing 48 h for wasps to emerge, figs from which at least one *P. minerva* had emerged were dissected to find and count any further *P. minerva* wasps remaining inside. This method provided counts of all *P. minerva* wasps in many figs, while not wasting large amounts of time (about 2 h per fig) dissecting the vast majority of figs (> 95%) that do not contain *P. minerva* wasps. It is, however, biased against detection of any figs that might contain only wingless males, which I address below (see results and discussion).

### Population proportions

For each fig, I recorded the number of *P. minerva* females, winged and wingless males and used these data to generate population totals and means for various metrics. In particular I calculated the overall proportion of winged males (pWM) and proportion of females dispersing unmated (pUF), i.e. those developing in a fig with no wingless males [8]. I then tested the morph ratio prediction of Hamilton’s model by comparing these proportions using a 2-sample test for equality of proportions (prop.test function in R). In addition, I made the same 2-sample comparison for each of the four sites. I then conducted separate 4-sample tests for equality of proportions to compare pWM and pUF across sites. Finally, I also calculated the overall population sex ratio (proportion males / all wasps) and tested if this differed from equality (0.5) again using the prop.test function.

### Testing for conditional morph and sex ratios

I then used the data to test if pWM decreases as the number of conspecific females in a fig increases, as predicted under conditional morph determination [23] and reported for some fig wasp species [27, 30, 38]. This used a generalised linear model (GLM) with binomial errors in R. In addition, I also conducted a similar analysis to test if the sex ratio increased with the number of wasps in a fig.

### Opportunity for local mate competition

Hamilton’s model assumes that females lay one egg per patch and is effectively a special case of Greeff’s [39] more complex model, which allows females to lay multiple eggs per fig and therefore creates the opportunity for local mate competition (LMC) between wingless males. The simpler model is justified if empirical data show that LMC occurs only rarely in natural situations (e.g. see Pienaar & Greeff [27]). Consequently, I calculated the frequency of figs with two or more wingless males, i.e. those where LMC could occur.

## Results

### Population data

**Fig. 3:**
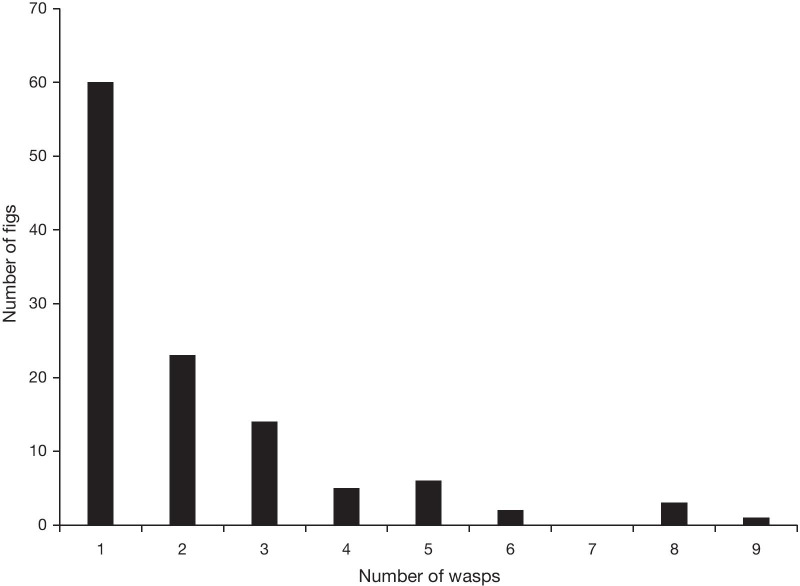
Numbers of *P. minerva* wasps in 114 occupied figs

*P. minerva* wasps only emerged from about 1 in 25 figs collected, resulting in 243 wasps from 114 figs from 44 trees at the four sites across Greater Sydney (Table 1). There was a mean of 2.13 wasps per fig and most (85%) occupied figs contained only 1–3 wasps (Fig. 3), suggesting that it is rare for females to lay more than 1 or 2 eggs per fig. The overall population sex ratio (proportion males = 0.33) was female-biased (χ^2^ = 27.671, df = 1, *p* = 1.438e-07).

**Table 1:** Site and population level sampling of *P. minerva* in Sydney

Site*	Figs	Trees	FU/F	+ M/M	Chi sq	p	Wasps	wasps/fig	SR
Balmoral Manly Penrith Wisemans	42 21 22 27	17 11 8 8	48/55 28/31 37/50 22/27	24/27 12/16 14/17 17/20	< 0.001 0.93 0.14 < 0.001	1 0.33 0.71 1	82 47 67 47	1.95 2.14 2.91 1.74	0.33 0.34 0.25 0.43
Sydney (all)	114	44	135/163	67/80	< 0.001	1	243	2.13	0.33

*Coordinates of sites: Balmoral (34.31S, 150.52E), Manly (33.81S, 151.29E), Penrith (33.75S, 150.69E), Wisemans Ferry (33.38S, 150.99E). *FM* females mated; *FU/F* females unmated/total females; *+ M/M* winged males/all males, *chisq* chi-squared value for 2-sample test for equality of proportions, *p* p-value; *wasps* total wasps; *wasps/fig* mean wasps per fig (see Fig. 3 for variation); *SR* sex ratio (proportion males/all wasps

### Testing for equal fitness of male morphs

At the population level, the proportion of winged males was 0.838 (67/80), which is not significantly different (χ^2^ < 0.001, df = 1, *p* = 1) from the estimated proportion (137/163 = 0.841) of females dispersing unmated, supporting the key quantitative prediction. In addition, pWM and pUF were not significantly different at any of the four sites when tested individually (Table 1) and neither pWM (χ^2^ < 1.47, df = 3, *p* = 0.69) nor pUF (χ^2^ < 4.76, df = 3, *p* = 0.19) differed significantly between sites.

However, my sampling may have underestimated the proportion of wingless males, due to the fact that, unlike winged males and females, they do not usually emerge from figs. While individual females, winged or wingless males may fail to develop fully or emerge successfully from their galls, these “failed” wasps are not expected to be biased towards a particular gender or morph. In contrast, amongst wasps that “succeed”, i.e. emerge from their galls and carry out normal subsequent behavior, there is a bias in my method against detecting wingless males. Figs were only dissected to reveal wasps remaining inside them if at least one wasp had emerged from the fig, and I had already excluded any figs from which a winged male or female may have already dispersed before collection (see methods). Since winged wasps emerge from figs to disperse, my counts of winged wasps should be accurate. However, most wingless males remain inside figs and so the data set may be missing rare figs containing only wingless male(s). This number of “missing wasps” can be estimated using the population data for mean numbers of wasps per fig, sex ratio and male morph ratio (Table 1; Additional file 1).

We can calculate the expected number of cases where there would be just one male (a singleton), either winged or wingless, by dividing the observed number (16) of singleton winged males by the observed proportion (51/64 = 0.80) of winged males in figs with two or more wasps. The justification for this is that we should have accurate estimates of both the number of singleton winged males, and the male morph ratio in samples where at least one winged wasp (male of female) was present. This yields an expected figure of 20.08 singleton males, implying 4.08 missed singleton wingless males. If we now consider the 21 figs with two wasps, only four have two males. Three cases have two winged males and one has a winged and a wingless male. The probabilities of different male combinations are: two winged (0.635), one of each (0.324), and two wingless males (0.041). It is therefore highly unlikely that any cases of two wingless males were missed, but we can include a correction factor of 0.041 × 4x2 = 0.38, to yield a total of 4.46 missing wingless males. By extension, a group consisting of only three wingless males is even more unlikely, and there are no cases of three or more males (of any type) at all in the empirical data, so I ignore this trivial possibility. If we now calculate the corrected proportion of winged males 67/84.46 = 0.793, this remains not significantly different (χ^2^ = 0.57257, df = 1, *p* = 0.4492) from the proportion of females dispersing unmated (0.841).

### Testing for conditional morph and sex ratios

**Fig. 4:**
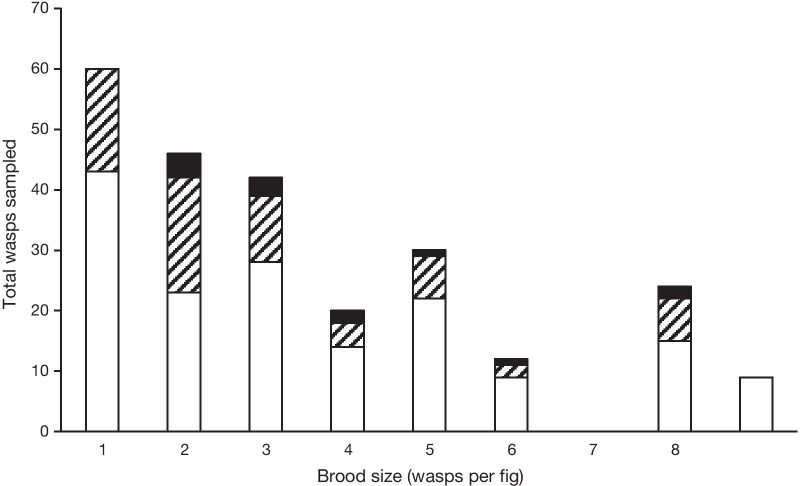
Numbers of females (white bars), winged males (hatched) and wingless males (black) in 114 figs with different numbers of wasps. NB singleton wingless males are not detectable with my sampling method—see text for further details

Neither the likelihood of being a winged male (*z* =  −  0.27; *P* = 0.79; d.f. = 1, 58) nor the sex ratio (*z* =  − 1.24; *P* = 0.22; d.f. = 1, 12) changed significantly as the number of wasps in a fig increased (Fig. 4). Hence, there is no evidence that either of these binomial variables are adjusted adaptively according to fig contents (Fig. 4).

### Opportunity for local mate competition

A total of 13 wingless males was recorded. In 11 figs there was only one wingless male, while one fig contained two wingless males. Consequently, there is very little opportunity for local mate competition between wingless males in the same fig.

## Discussion

This is the first detailed single species study of natural morph ratios in a fig wasp showing extreme male dimorphism (Fig. 1). Overall, the results are consistent with Hamilton’s [8] model that proposed morph ratios determined by frequency-dependent selection on their respective mating opportunities. I found that the proportion of *P. minerva* winged males (0.84) was not significantly different from the proportion of females dispersing unmated (0.84 or 0.79 corrected for sampling bias). This supports the hypothesis that sexual selection on mating opportunities defines the equilibrium proportion of winged males at the population level. In addition, the proportion of winged males is not correlated with the number of wasps in a fig (brood size). This is consistent with simple Mendelian segregation of alleles, but not with morph determination that is conditional on mating opportunities in the local patch (fig), as seen in some other fig wasps, where the proportion of wingless males increases with the number of wasps in a Fig. [23, 27]. Hence male morph proportions in *P. minerva* appear to be set by their average mating success at the population level, but not adjusted more finely to patch level opportunities.

The *P. minerva* population level data are consistent with Hamilton’s simple model of morph proportions [8]. However, various details of male behavior could decrease the reproductive value of wingless males relative to winged males and therefore select for an increased proportion of winged males. First, local mate competition between wingless males in the same fig could reduce their reproductive value and select for a higher proportion of winged males [39, 40]. However, in this study the average number of *P. minerva* wasps per fig was very low (2.13; Table 1) and only one fig contained more than a single wingless male, so local mate competition can be no more than a trivial force. Second, some females may disperse unmated from patches with wingless males, especially if there is lethal fighting between males [24, 40]. Fighting occurs between wingless males of some wing-dimorphic species [8], but not *P. minerva* whose wingless males (Fig. 1) lack the large jaws and armour of fighting fig wasps [25]. Moreover, they also lack the opportunity to fight as they so rarely co-occur with other wingless males. Third, winged males might sometimes mate within their natal fig with females [24, 40, 41], usurping some mating opportunities assumed to be taken by wingless males. This is harder to dismiss, but I found no evidence for it in the current study, where all winged males either emerged from figs or (only one) were unhatched from their galls. In contrast, wingless males emerge before winged males and females and have been observed to bite a small hole into galls containing females and crawl inside them to mate with the female inside the gall [24]. Consequently, the known biology of *P. minerva* does not suggest that any of these factors are significant.

I found no evidence that morph determination was conditional upon the number of wasps developing per fig. This contrasts with studies of *Otitesella* wasps [21, 27] that have no winged males but two wingless morphs (Fig. 2), where the proportion of the disperser morph decreases as the number of wasps in a fig increases. As the number of females in a fig increases, so do the mating opportunities for resident rather than dispersing males, and *Otitesella* wasps show fine-grained adaptation of morph ratios to local patch conditions [21, 27]. Such conditional morph determination allows more precise adaptation to mating conditions in the local patch and is also shown in *Rhizoglyphus echinopus* mites, which have morph proportions determined by colony size [18]. Such precise adaptation is not shown by *P. minerva* and is not possible under simple genetic control. Consequently, there seem to be at least two different mechanisms underlying male dimorphism in fig wasps. Male dimorphism is extreme in *P. minerva* [42]*,* with major differences in morph size, appendages, and body colour, as well as the most obvious difference of wings (Fig. 1). In contrast, the differences between the wingless *digitata* and *religiosa* morphs of *Otitesella* wasps (Fig. 2), while still striking, are more modest. In particular, as both morphs lack wings, even the dispersers are unlikely to travel beyond one or more branches on the same tree, so local (tree level) conditions are relevant to the dispersers as well as the non-dispersers. It is therefore not so surprising that a different mode of morph determination operates in these two cases, even though they have several aspects of selection in common.

An interesting case that falls between the *Pseudidarnes* and *Otitesella* examples of male polymorphism involves a fig wasp from a third different subfamily of wasps (Epichrysomallinae). *Sycobia* sp. has both winged and wingless males, but apart from the lack of wings and slightly smaller eyes in the wingless morph, the two morphs are similar in size and general appearance [38]. Niu et al. [38] found that the proportion of wingless males decreased with brood size, suggesting a conditional strategy. However, they confined females in small bags with figs and obtained brood sizes of up to 400 wasps per fig with a mean of over 100. It seems likely that the changes in morph ratio with brood size occurred in very large broods that would not occur under natural conditions. No data were presented on natural morph ratios or brood sizes, but for context the species mean brood size for the nine male-dimorphic fig wasp species in a comparative analysis was just seven [24].

Although my analyses here show that *P. minerva* morph ratios are not conditional on brood size, I did not test directly for genetic morph determination—an obvious next step. The male morphs produced by individual *P. minerva* females could be compared with the predictions of a single locus model. Here, we assume that the single locus controls a wing polymorphism that is male-limited; i.e. it only influences male offspring while females are always winged. This is the assumption used in the theoretical models of both Hamilton [8] and Greeff [34]. With the winged ( +) allele at a frequency of 0.8 and the wingless (−) allele at 0.2 there should be three female genotypes with frequencies of 0.64 (+ +), 0.32 (+ −) and 0.04 (−). In principle, a manipulative experiment could be used to produce arrays of offspring from females to compare with these expectations [38]. However, the logistics are challenging. First, the intricate life cycle requires females to be given access to figs at the right stage of development that have not been exposed to other conspecific wasps. Second, the figs must then be kept free from other conspecific wasps that might lay eggs, but able to ripen normally on the tree. Third, females seem to lay only one or two eggs per fig (Table 1), so many figs would probably be needed per female to get suitable offspring numbers, and there is a risk of females laying unusually high numbers of eggs per fig [38], as discussed above. An alternative would be to use molecular markers, such as microsatellites, to reveal parentage of unmanipulated wasps. This approach has been used successfully on other non-pollinating fig wasps to reveal the number of females laying eggs per fig and the number of offspring laid in a fig per mother [43, 44]. However, it rarely recovered offspring from the same female in different unmanipulated figs, presumably due to the dilution effect of large numbers of figs per tree and the option for females to visit many trees [43, 44]. Perhaps the best approach would be to combine manipulations and microsatellites to try to obtain offspring arrays from some females confined in bags around figs, then screen all emerging males genetically to identify brothers. An alternative approach is to use whole-genome sequencing of winged and wingless males and bioinformatics to search for consistent genetic differences between them [45]. However, while this may be good at revealing various genes that are differentially expressed and involved in traits such as eye and wing formation [38], these are unlikely to be the ones involved in a primary morph-determining signal.

Previous analyses of natural morph proportions in wing-dimorphic fig wasps [8, 24] have suffered from low sample size per species and/or data that are the by-product of other studies with different aims. In the current study, I sampled with the aim of estimating population averages for the two key variables (proportions of winged males and unmated females) for *P. minerva.* Hence the data derive from a small number of figs from each of a large number of trees, whereas most previous studies sampled data from many figs from only a few trees. Similarly, my samples were accumulated over many sampling sessions across four sites and two years, whereas many studies involve only one site and one or two sampling periods. The sampling of the current study is therefore better suited to estimating population averages for fig wasps whose abundance is highly variable in space and time [8, 27]. It is further possible that morph ratios and mating opportunities may vary across the canopy of large trees or throughout the year. These issues have yet to be investigated in any fig wasp studies, but could be an avenue for further research. Nevertheless, the year-round fruiting of this system (see methods) and fact that many study trees were small, allowing most or all of the canopy to be accessed (see methods) suggest that these may not be major factors.

While sampling was well-suited to the key questions, I also incorporated a shortcut that introduces sampling bias—only figs from which at least one wasp exited were dissected to reveal any further wasps within. Since wingless males rarely exit figs, this method could miss some figs that contain only a wingless male, or a rare, winged wasp that failed to exit successfully. I used the population data to infer the existence of four such “missing wingless males”, which is conservative since I ignored the possibility of missing winged males (or females). Applying this correction decreases the proportion of winged males from 0.84 to 0.79, but this remains not significantly different to the proportion of unmated females (0.84). Given that only about one in 25 figs contain *P. minerva* and dissection of one fig takes about two hours, it would have required an extra 5000 h of microscopy to dissect all figs sampled. Interestingly, we obtained a similar estimate of the proportion of winged males (0.77) using data collected as part of a fig wasp community ecology study in Melbourne some 25 years ago [35].

In contrast, the estimated proportion of females dispersing unmated was much lower in the earlier study (0.42) than observed here (0.83). Hence the frequencies of unmated females and winged males were significantly different and provide further evidence for lack of conditional adjustment of morph ratios to local mating opportunities. The earlier study involved figs from only four crops of fruit at a single site and was conducted outside the natural range of the host plant and its associated wasps. Surprisingly, a far higher proportion (95/145 = 0.66) of figs were occupied by *P. minerva*, which could indicate that the species is much more abundant in Melbourne, perhaps due to fewer competitor species outside its native range. Interestingly, high *P. minerva* abundance has also been reported for an introduced population in New Zealand [46]. However, several common fig wasp species from Sydney are also found in Melbourne [35], where the host plant is now well-established and common. I suspect that in studying only four fruit crops at one site we happened to sample crops where *P. minerva* was unusually abundant. In this context Hamilton [8] hypothesized that male wing-dimorphism might also be associated with high variability in wasp abundance, i.e. high variation in mating opportunities for winged and wingless males, and Pienaar & Greeff [21] also noted that such variation is common in fig wasps. The genus *Pseudidarnes* was recently revised by Farache et al. [42], but has received little ecological or behavioural study. The only population data for congeneric species are for *P. cooki*, which is associated with another Australian fig species, *F. obliqua*. Segar and Cook [47] analyzed data from 11 different sites and found it occurred in only 1 in 20 figs with rarely more than four wasps in the same fig. These population metrics are very similar to those reported for *P. minerva* in the current study (Table 1) and suggest that the high abundances of *P. minerva* reported outside its natural range may be atypical. To date, wingless males are not known for *P. cooki*, but it seems likely that they may exist.

## Conclusions

This detailed study of a fig wasp species with male wing-dimorphism supports the hypothesis that the frequencies of winged and wingless males are maintained by sexual selection on their mating opportunities. As the density of wasps increases, there are more mating opportunities for wingless males inside figs and fewer females disperse unmated. Those females emerging unmated (from figs without wingless males) are able to mate with winged males after dispersal. Consequently, selection should adjust the frequency of the winged morph to equal the proportion of females dispersing unmated, and we observe a very close correspondence of these two values in *P. minerva*. However, there is no correlation between morph proportions and the number of wasps per fig, suggesting absence of the conditional morph determination seen in some *Otitesella* fig wasps that have resident and disperser wingless male morphs [21, 27]. This means that morph proportions in *P. minerva* are adjusted to average mating success at the population level, but not at the patch (fig) level. Such coarse-grained adaptive fit is consistent with a simple Mendelian strategy, which now requires more direct genetic testing. Both wing-dimorphism and polymorphism amongst wingless males are common in fig wasps [8, 23, 24, 31] and great diversity can be found even within and across species from the one genus *Philotrypesis,* revealing considerable evolutionary lability [31]. Studies taking a phylogenetic perspective have tended to emphasize the repeated evolution of wingless male forms from winged males [23, 24, 31], although the re-emergence of winged males from wingless males has also been proposed for one lineage [32]. Regardless of the evolutionary polarity of such changes, detailed studies of other genera and species would be very interesting to probe whether conditional morph determination is linked particularly to cases with multiple wingless morphs, and genetic control to cases with wing-dimorphism.

## Supplementary Information

**Additional file 1. **Fig-level data on females and winged and wingless males.

## Data Availability

All data generated or analysed during this study are included in this published article, including Additional Information in Additional file 1.

## Abbreviations

LMC: Local mate competition
pWM: Proportion of winged males
pUF: Proportion of females dispersing unmated
