# Supplementary material for: Sexual selection on population-level mating opportunities drives morph ratios in a fig wasp with extreme male dimorphism
Source: BMC Ecol Evol. 2021 Sep 6;21:168. doi: 10.1186/s12862-021-01898-3 (PMC8422632; doi:10.1186/s12862-021-01898-3)
Supplement: Supplementary file 1 — Additional file 1. Fig-level data on females and winged and wingless males. [file 12862_2021_1898_MOESM1_ESM.pdf]

site	female	male1	male2	males	wasps
balmoral	1	0	0	0	1
balmoral	2	0	0	0	2
balmoral	1	0	0	0	1
balmoral	1	0	0	0	1
balmoral	1	0	0	0	1
balmoral	1	0	0	0	1
balmoral	0	2	0	2	2
balmoral	0	1	0	1	1
balmoral	3	1	0	1	4
balmoral	2	1	0	1	3
balmoral	1	0	0	0	1
balmoral	1	0	0	0	1
balmoral	2	0	0	0	2
balmoral	1	0	0	0	1
balmoral	0	1	0	1	1
balmoral	1	0	0	0	1
balmoral	1	0	0	0	1
balmoral	1	0	0	0	1
balmoral	1	0	0	0	1
balmoral	1	0	0	0	1
balmoral	0	2	0	2	2
balmoral	0	1	0	1	1
balmoral	4	3	1	4	8
balmoral	1	1	1	2	3
balmoral	1	0	0	0	1
balmoral	2	0	1	1	3
balmoral	1	0	0	0	1
balmoral	1	0	0	0	1
balmoral	1	0	0	0	1
balmoral	4	1	0	1	5
balmoral	1	0	0	0	1
balmoral	0	1	0	1	1
balmoral	1	1	0	1	2
balmoral	0	1	0	1	1
balmoral	3	2	0	2	5
balmoral	3	0	0	0	3
balmoral	2	1	0	1	3
balmoral	2	1	0	1	3
balmoral	1	1	0	1	2
balmoral	1	1	0	1	2
balmoral	3	1	0	1	4
balmoral	1	0	0	0	1
manly	9	0	0	0	9
manly	0	1	0	1	1
manly	1	0	0	0	1
manly	2	1	0	1	3
manly	1	0	0	0	1
manly	4	1	0	1	5
manly	1	0	1	1	2
manly	0	1	0	1	1
manly	1	0	1	1	2

manly	1	0	0	0	1
manly	3	1	0	1	4
manly	1	1	0	1	2
manly	1	0	0	0	1
manly	1	0	1	1	2
manly	0	1	0	1	1
manly	1	2	0	2	3
manly	1	0	0	0	1
manly	1	1	0	1	2
manly	0	1	0	1	1
manly	1	0	0	0	1
manly	0	1	1	2	2
manly	1	0	0	0	1
penrith	5	0	0	0	5
penrith	2	0	0	0	2
penrith	1	0	0	0	1
penrith	4	0	1	1	5
penrith	6	1	1	2	8
penrith	1	0	0	0	1
penrith	3	0	0	0	3
penrith	6	0	0	0	6
penrith	1	1	0	1	2
penrith	1	0	0	0	1
penrith	1	1	0	1	2
penrith	0	1	0	1	1
penrith	1	0	0	0	1
penrith	1	0	0	0	1
penrith	5	3	0	3	8
penrith	1	0	0	0	1
penrith	3	1	0	1	4
penrith	3	0	0	0	3
penrith	0	2	0	2	2
penrith	3	2	1	3	6
penrith	1	1	0	1	2
penrith	1	0	0	0	1
penrith	0	1	0	1	1
wisemans	1	0	0	0	1
wisemans	1	0	0	0	1
wisemans	1	0	0	0	1
wisemans	2	0	2	2	4
wisemans	0	1	0	1	1
wisemans	0	1	0	1	1
wisemans	1	1	0	1	2
wisemans	0	1	0	1	1
wisemans	1	0	0	0	1
wisemans	3	0	0	0	3
wisemans	1	2	0	2	3
wisemans	1	0	0	0	1
wisemans	1	0	0	0	1
wisemans	1	0	0	0	1
wisemans	1	1	0	1	2
wisemans	1	0	0	0	1

wisemans	2	3	0	3	5
wisemans	1	0	0	0	1
wisemans	2	0	0	0	2
wisemans	0	1	0	1	1
wisemans	1	1	0	1	2
wisemans	1	1	1	2	3
wisemans	1	1	0	1	2
wisemans	2	1	0	1	3
wisemans	1	0	0	0	1
wisemans	0	1	0	1	1
wisemans	0	1	0	1	1
